# Word length and frequency effects in natural Chinese reading: Evidence for character representations in lexical identification

**DOI:** 10.1177/17470218241281798

**Published:** 2024-10-06

**Authors:** Ying Fu, Simon P Liversedge, Xuejun Bai, Maleeha Moosa, Chuanli Zang

**Affiliations:** 1 Faculty of Psychology, Tianjin Normal University, Tianjin, China; 2 Department of Psychology, Yancheng Teachers University, Yancheng, China; 3 School of Psychology and Humanities, University of Central Lancashire, Preston, UK

**Keywords:** Word length, word frequency, visual complexity, Chinese reading, eye movements

## Abstract

Word length and frequency are two of the “big three” factors that affect eye movements in natural reading. Although these factors have been extensively investigated, all previous studies manipulating word length have been confounded with changes in visual complexity (longer words have more letters and are more visually complex). We controlled stroke complexity across one-character (short) and two-character (long) high- and low-frequency Chinese words (to avoid complexity confounds) and recorded readers’ eye movements during sentence reading. Both word length and frequency yielded strong main effects for fixation time measures. For saccadic targeting and skipping probability, word length effects, but not word frequency effects, occurred. Critically, the interaction was not significant regardless of stroke complexity, indicating that word length and frequency independently influence lexical identification and saccade target selection during Chinese reading. The results provide evidence for character-level representations during Chinese word recognition in natural reading.

## Introduction

Word identification is essential for sentence comprehension and lies at the heart of all current eye movement models. Word length and word frequency are fundamental lexical characteristics that affect word identification and both affect eye movement control during natural reading of alphabetic languages (Clifton et al., 2016; Liversedge & Findlay, 2000; Rayner, 1998, 2009) and Chinese ( X.Li et al., 2015). Short words are associated with shorter fixation durations and increased skipping probability (SP) relative to long words (Joseph et al., 2009; Ma et al., 2019; Rayner et al., 1996; Zang et al., 2018). High-frequency words are fixated for less time than low-frequency words (Cui et al., 2021; Inhoff & Rayner, 1986; Kliegl et al., 2004; Yan et al., 2006). Although numerous studies have separately examined word length and word frequency effects on eye movements during reading, the joint effects of these two factors are still not well understood. In alphabetic languages, only a small number of studies have independently manipulated the two variables to assess whether their effects are additive or interactive and these have produced mixed results. In Chinese, however, how these two variables jointly affect eye movement control during reading remains unexplored. This was the purpose of the present study. This line of work is important because the joint constraint of word frequency and length on eye movements is of particular significance to a current model of eye movement control during Chinese reading, namely the Chinese Reading Model (X. Li & Pollatsek, 2020).

In alphabetic languages, the joint effect of word length and frequency has been examined using tachistoscopic techniques (measuring minimal exposure time required for identifying a word), naming, and lexical decision tasks (see Barton et al., 2014 for a review). McGinnies et al. (1952) detected an interactive effect using the tachistoscopic technique such that a word length effect was found only for low- but not high-frequency words (see also Adis-Castro & Postman, 1957). However, Doggett and Richards (1975) using a similar technique failed to find an interaction between the two variables. In naming and lexical decision tasks, some researchers have reported stronger word length effects for low- than high-frequency words (Weekes, 1997; Balota et al., 2004; Lee, 1999; Yap & Balota, 2009), while some other studies showed additive effects of length and frequency (New et al., 2006; O’Regan & Jacobs,1992). For example, based on a large dataset of words from the English Lexicon Project (Balota et al., 2002), New et al. analysed the lexical decision data and observed significant word frequency effects across the word length spectrum (from 3–4 letters to 12–13 letters). Importantly, they found effects of word length independent of word frequency.

Several studies have shown that word length and frequency interactively affect eye movement control during reading (Bertram & Hyönä, 2003; Harvey et al., 2017; Kaakinen & Hyöna, 2010; Pollatsek et al., 2008; Schroyens et al., 1999). For example, Schroyens et al. (1999) found increased word frequency effects for five-letter words compared with three-letter words when native Dutch speakers read word triads. Pollatsek et al. (2008) examined the effects of varying the frequency and length of an adjective followed by a noun (Experiment 1) during sentence reading and showed a frequency effect on gaze duration (GD) that was significant for seven- to nine-letter words, but not for three- to four-letter words. Using the same materials, Harvey et al. (2017) found similar results when participants read both horizontally scrolling and standard static text. Bertram and Hyönä (2003, Experiment 2) manipulated (whole) word frequency for short (⩽8 letters) and long (⩾12 letters) Finnish compound words and found a weak interaction between word length and frequency in participants, but not item analyses of GD.

In contrast, other studies have shown that word length and frequency additively affect eye movements during reading (Rayner et al., 1996; Tiffin-Richards & Schroeder, 2015). Rayner et al. (1996) monitored participants’ eye movements when they read short narratives with an equal number of high- or low-frequency 5-, 6-, 7-, 8-, 9-, and 10-letter target words. Results provided no evidence of interactive effects. Tiffin-Richards and Schroeder (2015) used age-appropriate word frequency stimuli for children to investigate the effects of frequency and length (three to four letters or eight to nine letters) on both children’s and adults’ eye movements in reading. Although they found a stronger word length effect for infrequent than frequent words for children, there was no reliable interaction for adults. One likely reason for this inconsistency is variability in word length manipulations across studies (Short words: 3 letters, 3–4 letters or ⩽ 8 letters; Long words: 5 letters, 7–9 letters, 8–9 letters or ⩾12 letters), and that the relationship between frequency and length, itself, changes across that range of word lengths. The variability in word length criteria across studies is very important because word length is correlated with word frequency (Rayner & Duffy, 1986).

Unlike alphabetic languages, Chinese text comprises equally sized characters and there are no spaces to separate words. Also, the variance in word length in Chinese is dramatically reduced relative to alphabetic languages. Actually, in written Chinese text, more than 90% of words are one or two characters long (see Zang, Wang, et al., 2016). Also, the frequency for each level of word length is very comparable—the relationship between word length and frequency is, thus, more stable. Hence, it is relatively easy to assess the effects of the frequency of one- or two-character words in Chinese without concerns as to confounds between word length and word frequency. However, as noted, hitherto, no research has directly varied the word length and frequency of Chinese words either presented in isolation in word recognition tasks or embedded in sentences to investigate their joint effects on eye movements during natural reading. Therefore, the first theoretical question we will explore concerns whether word length and frequency additively or interactively constrain processing during Chinese reading.

By investigating word length and frequency effects in Chinese reading, we were also able to examine a second important theoretical question. Of course, in alphabetic languages, word length manipulations necessarily involve the selection of words with more letters (long words) or fewer letters (short words; e.g., Joseph et al., 2009). This means that in every study of alphabetic word length effects that has been conducted to date, manipulations of word length have been fundamentally confounded with a word’s visual complexity. To be clear, on average, a word with more letters is more visually complex than a word with fewer letters.^1^ To date, there has never been an investigation of word length effects in reading that has tackled this confound. One of the primary drivers for the present study was to carry out, for the first time, an investigation of the effects of word length and frequency on eye movements during reading in the absence of visual complexity confounds. Moreover, as will be apparent, we designed our experiment to directly assess the influences of word length and frequency under different conditions of visual complexity. We consider this to be a novel and theoretically interesting question that it is possible to investigate in Chinese reading. As is well documented, Chinese characters are formed from strokes, and a generally well-accepted metric of the visual complexity of a Chinese word is its stroke complexity (X. Li et al., 2014; Ma & Li, 2015). The visual complexity of Chinese words can vary independent of their length. For example, the word “人” (means person) and the word “霾” (means haze); both are one-character words; however, the word “霾” comprises 22 strokes and is much more visually complex than the word “人” which comprises just two strokes. Given this characteristic of the Chinese orthography, it is a perfect candidate language in which to examine the effects of word length in the absence of confounding changes in visual complexity. Unfortunately, in all previous studies examining word length effects in Chinese reading, visual complexity of target words was not considered (X. Li et al., 2011; S. Li et al., 2018), nor experimentally controlled (X. Li & Shen, 2013; Ma et al., 2019; Zang et al., 2018). This is something of an oversight, in that visual complexity affects both lexical identification and saccade target selection during reading when the word length is matched. Words with low visual complexity take less time to process and are skipped more often and targeted closer to their centre than words with high visual complexity (Liversedge et al., 2014; Ma & Li., 2015; Zang, Zhang, et al., 2016; Zang et al., 2018). Thus, as noted earlier, the second important theoretical issue we investigated in the current experiment was whether word length effects occurred during word identification during natural reading in the absence of concurrent corresponding changes in visual complexity. Furthermore, we examined the effects of frequency and length for words differing in visual complexity.

These two theoretical issues are of particular significance to the Chinese Reading Model (CRM), a recent formal computational model proposed by X. Li and Pollatsek (2020). Here we focus on the CRM because it is the first, and only current, integrated model of word processing and eye movement control in Chinese reading and it provides a novel and unique account of how word identification and word segmentation occur during such processing. According to the CRM, all the characters that fall within the perceptual span are visually processed in parallel and the corresponding characters represented at the character level within the mental lexicon are activated. Beyond this, word units corresponding to the activated characters become activated accordingly, and these word units compete with each other to be identified. The outcome of the identification process is critically dependent on the lexical characteristics of word length and frequency. When a word unit wins the lexical competition, it is simultaneously segmented and identified from the upcoming string of characters. That is, the identified word is determinant with respect to decisions concerning eye movement control, specifically when and where to move the eyes.

In the CRM, word frequency directly affects the activation of word units and the activation for high-frequency words accrues faster than activation for low-frequency words. Therefore, readers take longer to process and identify low than high-frequency words. Furthermore, the CRM (and models of word identification more generally) specify that words with more characters take longer to identify than shorter words because there is more content in the word to process. These basic predictions are straightforward. However, the precise interactive pattern that we might expect on the basis of the CRM is less immediately obvious. In respect of this, it is important to note that there are many more two-character than one-character words in Chinese (with an approximate ratio of 7:1). Thus, the set of activated candidates will almost certainly be greater for two- than one-character words.

There could be two possible consequences of this situation. First, reduced numbers of one compared with two-character activated words would result in correspondingly reduced levels of overall activation in the mental lexicon, meaning that the process of word identification overall would occur less efficiently (rapidly) for one- than two-character words. In turn, this would imply that the magnitude of any frequency effects is likely to be reduced for long than for short words. An alternative possibility is that the number of candidates that are activated will be substantially reduced for one- than two-character words. A reduced number of activated candidates might lead to a more efficient resolution process which might consequently lead to frequency effects of smaller magnitude for short than long words. Without direct generation of predictions through simulations, it is difficult to know which of these patterns the CRM might predict. However, what we can be sure of is that regardless of the precise pattern of effects, the CRM does stipulate that the influence of frequency and word length will be interactive.

X. Li and Pollatsek (2020) successfully simulated the effects of word length, and separately, the effects of word frequency based on the eye movement data from X. Li et al. (2011). However, to reiterate, no study has examined the combined effects of the two. Accordingly, in the present experiment, we orthogonally manipulated word length (one-character words or two-character words) and word frequency (high frequency or low frequency) to assess their joint effect in Chinese reading. Furthermore, to determine whether, and how, the effects of word length and frequency were modulated by visual complexity, we included two different two-character word conditions for words that were high and words that were low frequency. Specifically, we included a two-character word condition in which the word’s total stroke number matched the one-character word, as well as a two-character word condition in which the stroke number of the word’s first character matched that of the one-character word. In this way, we could be sure that any processing differences we obtained between short and long words (and in relation to frequency) arose due to effects of word length and not differences in visual complexity as measured across the entire two-character word, or in relation to its initial character.

The present study was a 2 (word frequency: high, low) × 3 (word length: one-character word, two-character word with its total stroke number equal to the one-character word, two-character word with the stroke number of its first character matched to that of the one-character word) repeated measures design. We based our predictions on previous studies (Cui et al., 2021; Ma et al., 2019; Wei et al., 2013; Yan et al., 2006; Zang et al., 2018) and the CRM. First, we predicted the main effects of word length and word frequency, with readers taking longer to process long or low-frequency words than short- or high-frequency words. Second, we predicted that effects of word length would occur independent of visual complexity due to the two characters of two-character words delivering increased lexical activation relative to that delivered by a single-character word regardless of visual complexity. To be clear, we anticipated activation metrics at the word level themselves to be determined by character-level activation. Third, we predicted an interaction between word length and frequency. On the basis of the specifications of the CRM, it is possible that frequency effects may be greater in magnitude for long than short words, or vice versa. Regardless, interactive effects should occur and these should not be modulated by visual complexity.

## Method

### Participants

A total of 72 native Chinese speakers (average years = 23.5 years, range 19–35 years, *SD* = 2.5 years, 11 males, 61 females) with normal or corrected-to-normal vision from Tianjin Normal University participated in the experiment. We calculated effect sizes for studies reporting interactive effects between word length and frequency. The range of effect sizes for these studies ranged from 0.40 to 1.40 (Bertram & Hyönä, 2003: *d* = 0.55; Pollatsek et al., 2008: *d* = 1.40; Schroyens et al., 1999: *d* = 0.56; Kaakinen & Hyöna, 2010: *d* = 0.40; Harvey et al., 2017: *d* = 0.43). The average *d* value is 0.67. A power analysis was conducted using the PANGEA software developed by Westfall (2015), and at least 36 participants in total were required for 42 stimuli to allow for the detection of an effect of 0.67 with a power of 0.80. Our sample exceeded that required as a minimum.

### Materials and design

A total of 252 target words were selected from a database developed by Cai and Brysbaert (2010), creating a manipulation for word length and word frequency with 42 one-character high-frequency (1CW-HF) words, 42 one-character low-frequency (1CW-LF) words, 42 two-character high-frequency words with total stroke number equal to the one-character words (2CWT-HF), 42 two-character low-frequency words with total stroke number equal to the one-character words (2CWT-LF), 42 two-character high-frequency words with first character stroke number equal to the one-character words (2CWF-HF), and 42 two-character low-frequency words with first character stroke number equal to the one-character words (2CWF-LF). A total of 42 experimental sentence frames were created in total, and each sentence frame contained a target word from each of the six stimulus categories and was identical at least up to the target word (see Table 1).

**Table 1.** An example of experimental sentences (target words are in bold).

Condition	Sentence
1CW-HF	王亚娟在**洗**衣服的时候看到了赵团长。
1CW-LF	王亚娟在**晾**衣服的时候看到了赵团长。
2CWT-HF	王亚娟在**放下**衣服的时候看到了赵团长。
2CWT-LF	王亚娟在**批发**衣服的时候看到了赵团长。
2CWF-HF	王亚娟在**制作**衣服的时候看到了赵团长。
2CWF-LF	王亚娟在**赎回**衣服的时候看到了赵团长。

The translation for this sentence is *“Wang Yajuan spotted Captain Zhao while in the process of* ***washing/ drying/ putting down/ purchasing/ making/ trading*** *clothes.”*

The high-frequency words were in the range of 30–426 occurrences per million (*M* = 119) and the low-frequency words were in the range of 0.06–5 occurrences per million (*M* = 1.25). The difference between high- and low-frequency words was reliable, *F*(1,41) = 194, *p* < .001. However, there were no differences in frequency between words in the 1CW, the 2CWT, and the 2CWF conditions (all *F* < 1, *p* > .46). Furthermore, differences in the total stroke number for characters in the 1CW condition, the total stroke number in the 2CWT condition and the stroke number of the first character in the 2CWF condition approached significance, *F*(2, 40) = 3.64, *p* = .063. Planned comparisons showed there were no significant differences between any two of the three word length conditions (all *p* > .162). The characteristics of these stimuli are listed in Table 2.

**Table 2.** The characteristics of the experimental sentences and target words for each condition.

Condition		Stroke number	Frequency	Sentence naturalness	Predictability
1CW	HF	10.38(1.21)	129.81(97.25)	3.88(0.29)	0.02(0.01)
	LF	10.57(1.17)	1.42(0.99)	3.80(0.33)	0.00(0.00)
2CWT	HF	10.95(0.99)	124.02(99.83)	3.76(0.37)	0.00(0.00)
	LF	10.79(1.20)	1.27(0.92)	3.75(0.35)	0.00(0.01)
2CWF	HF	10.40(1.29)	104.26(86.87)	3.82(0.33)	0.02(0.01)
	LF	10.55(1.17)	1.06(0.81)	3.84(0.34)	0.00(0.01)

The stroke number of 2CWF is the stroke number of the first character.

Sentences were between 15 and 21 characters long (*M* = 18, *SD* = 1) and were rated for naturalness on a 5-point scale (5 = very natural) by participants who did not participate in the eye-tracking study. The mean naturalness score was 3.8 (*SD* = 0.3), with no differences for word length, word frequency, and their interaction (all *F* *<* 1.73, *p* > .184). Predictability norms from 15 additional participants confirmed that target words of each condition were unpredictable from sentence context (*M* = 0%, *SD* = 1%).

Six files were constructed with each file containing 42 sentences and conditions were rotated across files according to a Latin Square. Each participant read experimental sentences presented randomly from one of the six files with eight practice sentences at the beginning of the experiment. One-third of sentences were followed by a “yes” or “no” comprehension question.

### Apparatus and procedure

Eye movements were recorded via an SR Research EyeLink 1000 eye tracker at a sampling rate of 1,000 Hz. Viewing was binocular and movements of one eye were recorded. Participants were seated 65 cm from a 19-in. monitor (refresh rate of 120 Hz), and one Chinese character subtended approximately 1.1° of visual angle.

Participants were instructed to read the sentences naturally and carefully and responded to a comprehension question occasionally regarding the sentence they had just read. At the beginning of the experiment, participants were calibrated using a 3-point horizontal calibration grid, with an acceptance criterion of an average error below 0.25 degrees. A drift correction was implemented before each sentence and recalibrated as necessary. The experiment lasted approximately 15 min.

### Results

The overall comprehension rate was 95% (ranging from 86% to 100%) indicating that all participants read and fully understood the sentences. Fixation durations shorter than 80 ms or longer than 1,200 ms were deleted from the data set. Trials were removed if there was tracker loss, fewer than three fixations in total (0.03% of the data), and measures were above or below 3 SDs from each participant’s mean (1%). Analyses were carried out for the target word region. The following eye movement measures were computed: first fixation duration (FFD, duration of the first fixation on a word), single fixation duration (SFD, fixation duration when only one fixation was made), GD (sum of all first-pass fixations on a word before leaving it), total reading time (TRT, sum of all fixations), SP (the probability of skipping a word), mean landing position (MLP, measured by character), and launch site distance (LSD, position of the previous fixation, measured as the number of characters to the left of the target region). Means and standard deviations for the eye movement measures for target words are shown in Table 3.

**Table 3.** Means and standard deviations for all eye movement measures on the target words.

Condition		FFD	SFD	GD	TRT	SP	MLP	LSD
1CW	HF	241(38)	240(41)	248(44)	288(62)	0.49(0.22)	0.53(0.18)	1.39(0.53)
	LF	252(52)	255(59)	261(65)	302(91)	0.49(0.22)	0.47(0.17)	1.39(0.50)
2CWT	HF	237(41)	237(42)	277(78)	376(131)	0.21(0.21)	0.91(0.27)	1.45(0.56)
	LF	253(47)	252(53)	290(63)	404(99)	0.20(0.23)	0.89(0.25)	1.40(0.50)
2CWF	HF	238(32)	237(35)	267(54)	364(108)	0.17(0.18)	0.95(0.25)	1.38(0.43)
	LF	251(35)	255(43)	300(64)	405(115)	0.14(0.16)	0.93(0.24)	1.36(0.55)

FFD = first fixation duration; SFD = single fixation duration; GD = gaze duration; TRT = total reading time; SP = skipping probability; MLP = mean landing position; LSD = launch site distance.

Linear mixed-effects models (LMMs) were conducted using the lme4 package (Version 1.1-21) within the R 3.6.0 (R Development Core Team, 2019). As fixed factors, we included word length, word frequency, and their interaction. For all measures, models with maximum random effects structure (Barr et al., 2013) were conducted, allowing random intercepts and random slopes for both participants and items. If the maximum random model failed to converge, the model was trimmed by first removing correlations between variables, then random factors for items, and then participants until the model converged. Three contrasts were set up using the “contr.sdif ()” function from the MASS package: (1) 1CW versus 2CWT; (2) 1CW versus 2CWF; and (3) 2CWT versus 2CWF. The first two comparisons were conducted to assess effects of word frequency and word length when the two words were matched on their total visual complexity, or the complexity of the whole (one-character) word and the first character of the two-character word, respectively. The third comparison was conducted to evaluate whether differences in the visual complexity of the initial character of otherwise matched high- and low-frequency words might affect the degree to which they were skipped and how they are fixated (i.e., initial fixation positions on them. To increase the normality of the data, fixation times and landing position were log-transformed. Logistic LMMs were carried out for SP. Effects were taken to be statistically significant if the |*t|* or |*z|* value was greater than 1.96 (e.g., Drieghe et al., 2019). Fixed effect estimations for the eye movement measures are shown in Table 4.

**Table 4.** Results for linear mixed models for all eye movement measures on the target words.

Contrast	FFD				SFD				GD				TRT			
	*b*	CI	*SE*	*t*	*b*	CI	*SE*	*t*	*b*	CI	*SE*	*t*	*b*	CI	*SE*	*t*
Intercept	5.46	[5.44,5.48]	0.01	**438.11**	5.46	[5.44,5.49]	0.01	**418.98**	5.54	[5.51,5.58]	0.02	**326.71**	5.75	[5.70,5.80]	0.03	**228.61**
1CW versus 2CWT	−0.00	[−0.04,0.03]	0.02	−0.20	−0.00	[−0.04,0.03]	0.02	−0.29	0.09	[0.05,0.13]	0.02	**4.51**	0.24	[0.19,0.29]	0.02	**9.89**
1CW versus 2CWF	0.00	[−0.03,0.03]	0.02	0.04	0.01	[−0.03,0.04]	0.02	0.45	0.10	[0.07,0.14]	0.02	**5.26**	0.24	[0.19,0.28]	0.02	**9.97**
2CWT versus 2CWF	0.00	[−0.02,0.03]	0.01	0.27	0.01	[−0.02,0.04]	0.02	0.80	0.01	[−0.02,0.05]	0.02	0.77	−0.00	[−0.04,0.04]	0.02	−0.05
Frequency	0.04	[0.02,0.07]	0.01	**3.32**	0.05	[0.02,0.08]	0.01	**3.64**	0.07	[0.04,0.10]	0.02	**4.24**	0.08	[0.04,0.12]	0.02	**4.22**
1CW versus 2CWT × Frequency	0.01	[−0.05,0.08]	0.03	0.45	0.01	[−0.06,0.07]	0.03	0.21	0.02	[−0.06,0.10]	0.04	0.41	0.07	[−0.03,0.16]	0.05	1.41
1CW versus 2CWF × Frequency	−0.00	[−0.07,0.06]	0.03	−0.12	0.00	[−0.06,0.07]	0.03	0.13	0.06	[−0.02,0.14]	0.04	1.49	0.09	[−0.00,0.18]	0.05	1.89
2CWT versus 2CWF × Frequency	−0.02	[−0.07,0.04]	0.03	−0.65	−0.00	[−0.06,0.06]	0.03	−0.09	0.04	[−0.03,0.11]	0.04	1.20	0.02	[−0.06,0.11]	0.04	0.51
Contrast	SP				MLP				LSD							
	*b*	*CI*	*SE*	*z*	*b*	*CI*	*SE*	*t*	*b*	*CI*	*SE*	*t*				
Intercept	−1.20	[−1.44,–0.97]	0.12	**–10.00**	−0.59	[−0.64,–0.54]	0.03	**–22.57**	−0.02	[−0.09,0.05]	0.04	−0.68				
1CW versus 2CWT	−1.50	[−1.72,–1.28]	0.11	**–13.66**	0.54	[0.44,0.65]	0.06	**9.79**	0.03	[−0.06,0.11]	0.04	0.65				
1CW versus 2CWF	−1.90	[−2.14,–1.67]	0.12	**–16.13**	0.60	[0.49,0.71]	0.05	**10.95**	0.00	[−0.08,0.09]	0.04	0.03				
2CWT versus 2CWF	−0.40	[−0.65,–0.16]	0.12	**–3.27**	0.06	[−0.04,0.15]	0.05	1.18	−0.03	[−0.11,0.06]	0.04	−0.62				
Frequency	−0.11	[−0.29,0.08]	0.09	−1.13	−0.08	[−0.16,0.01]	0.04	–1.81	−0.03	[−0.10,0.04]	0.04	−0.96				
1CW versus 2CWT × Frequency	−0.06	[−0.48,0.36]	0.21	−0.30	0.18	[−0.04,0.40]	0.11	1.60	−0.02	[−0.19,0.15]	0.09	−0.24				
1CW versus 2CWF × Frequency	−0.26	[−0.71,0.19]	0.23	−1.14	0.18	[−0.04,0.39]	0.11	1.61	−0.01	[−0.18,0.17]	0.09	−0.06				
2CWT versus 2CWF × Frequency	−0.20	[−0.68,0.29]	0.25	−0.81	−0.00	[−0.19,0.19]	0.10	−0.01	0.02	[−0.16,0.19]	0.09	0.19				

Significant items are presented in bold, *b* = regression coefficient; *CI* = confidence interval. FFD = first fixation duration; SFD = single fixation duration; GD = gaze duration; TRT = total reading time; SP = skipping probability; MLP = mean landing position; LSD = launch site distance. The model for FFD, GD, TRT, SP, MLP, and LSD following trimming was as follows: depvar ~ wordlength* wordfrequency + (1|pp) + (1|item). The model for SFD was as follows: depvar ~ wordlength* wordfrequency + (1|pp).

#### Fixation times

There was no effect of word length on FFD and SFD (all |*t*| < 0.46). However, GD and TRT were significantly longer for 2CWT and 2CWF targets than 1CW targets (all *t* > 4.50), indicating that the effect of word length on fixation duration was mainly driven by refixations (see also X. Li et al., 2011; X. Li & Shen, 2013; Zang et al., 2018). There were no reliable differences between 2CWT and 2CWF targets on all fixation measures, suggesting that effects on fixation measures were not driven by the modest visual complexity differences between the two types of two-character words. Consistent with established findings in Chinese (Cui et al., 2021; Wei et al., 2013; Yan et al., 2006), we found a robust word frequency effect for all fixation time measures, with low-frequency words associated with longer times than high-frequency words (all *t* > 3.31).

Importantly, the interactions between word length and frequency for fixation time measures were not reliable, neither when the total stroke number of the target, nor when the stroke number of the initial character was matched with that of the single character target (all |*t|* < 1.90). This finding indicates that word length and word frequency independently affect fixation times during Chinese reading and these influences are not modulated by the visual complexity of the initial character or the whole two-character word. Indeed, the interactive effect between the two two-character words (2CWT vs. 2CWF) and frequency was also not significant across all fixation time measures (all |*t*| < 1.21). Again, this demonstrates that visual complexity did not modulate the effects of frequency and length in the processing of two-character words during Chinese reading.

To further examine the null interaction, we conducted Bayes factor analyses for linear mixed models (Morey et al., 2018) for fixation time measures. Bayes factors were calculated for the full model (*BF*_Full_) and the model only including the main effects (*BF*_Main_). We evaluated the non-significant interaction by comparing these two models (*BF* = *BF*_Full_/*BF*_Main_) and used the default scale prior (*r* = .5) and 100,000 Monte Carlo iterations of the BayesFactor package in R for reading time measures. The results showed that all Bayes factors were less than 1. A sensitivity analysis with different priors (i.e., 0.2, 0.3, 0.4, 0.5, 0.6, 0.7, and 0.8) showed consistent results (all *BF*s﹤1).

#### Word skipping

For word skipping, the 1CW targets were skipped more often than both forms of the two-character targets (all |*z|* > 13.65). Note that here the effect of word length was observed independent of complexity confounds. There was an effect of visual complexity between 2CWT and 2CWF targets as 2CWT targets were skipped more frequently than 2CWF targets (*|z|* = –3.27*).* These findings indicate that both word length and visual complexity impact SP in Chinese reading and the independent effect associated with each of these variables can be observed in a non-confounded situation. Perhaps the most important point to note in this regard is that these results show (for the first time) that it is possible to observe robust influences of word length in the absence of changes in visual complexity, and therefore, it seems likely that word length, per se, is a factor of influence in lexical identification during Chinese reading. There was no significant difference in SP between high- and low-frequency words (*z* = –1.13), nor were there any reliable interactions (all |*z*|< 1.15). As with the reading time results, word length and word frequency exerted independent effects on SP and these effects occurred regardless of visual complexity.

#### MLP and launch site

MLP was significantly further to the right for 2CWT targets and 2CWF targets than 1CW targets (all *t* > 9.78). The difference between 2CWT and 2CWF was not significant. This pattern of results suggests that initial saccadic targeting to upcoming words was primarily affected by the length rather than the visual complexity of their constituent characters. There was a non-significant effect of frequency (*t* = –1.81). Numerically, MLPs were slightly more rightward for high (*M* = 0.80) than low-frequency words (*M* = 0.76). The robust frequency effects on reading time measures, alongside the absence of such effects for the skipping and landing position measures suggests that this linguistic factor is a primary influence on lexical identification but not saccadic targeting during Chinese reading (i.e., frequency primarily affects decisions of when but not where to move the eyes). Neither the interactions between word length and word frequency nor the interaction between the two types of two-character words (2CWT vs. 2CWF) and frequency were significant in the landing position analyses (all *t* < 1.62). Again, these results provide no evidence for interactive influences of word length and frequency, nor was there any suggestion of a modulatory influence of visual complexity. Finally, unsurprisingly, our manipulations produced no significant effects for saccade launch sites (*|t|* < 0.97), indicating that the landing position effects did not arise due to launch site differences.^2^

## Discussion

In the present study, we monitored the eye movements of native Chinese readers when they read sentences containing target words that were orthogonally manipulated for word length (one-character word, two-character word with its total stroke number equal to the one-character word, two-character word with the stroke number of its first character matched to that of the one-character word) and word frequency (high and low). Our objectives with these manipulations were to investigate whether word length effects occurred in the absence of visual complexity confounds, assess whether effects of word length and frequency on eye movements were additive or interactive, and assess whether the effects of these two factors were modulated by visual complexity during Chinese reading. Our results revealed that word length, in the absence of complexity confounds, does affect lexical identification and saccade target selection during Chinese reading. Specifically, long words are associated with longer GDs and total fixation durations (though not longer FFD or SFD), were skipped less often, and were initially fixated further into the word, than was the case for short words. These results are consistent with previous studies (e.g., Zang et al., 2018) and demonstrate that a word’s length is a fundamental characteristic independent of any concomitant factor pertaining to its visual complexity. In addition, readers were quicker to process high- than low-frequency words due to their greater familiarity with the former. Importantly, our findings provided no evidence for interactions between word length and frequency on any eye movement measures, and again, this held regardless of visual complexity. Recall the mixed results in alphabetic languages for word length and frequency manipulations. Some studies showed main effects (Rayner et al., 1996; Tiffin-Richards & Schroeder, 2015) and others interactions (Bertram & Hyönä, 2003; Harvey et al., 2017; Kaakinen & Hyöna, 2010; Pollatsek et al., 2008; Schroyens et al., 1999). It seems that for complexity controlled Chinese words that are one or two characters long (i.e., the vast majority of Chinese words), the magnitude of frequency effects appears comparable.

In the next section, we will consider the implications of our findings in relation to the CRM (X. Li & Pollatsek, 2020). Recall the assumptions of the CRM. Readers process all characters within the perceptual span and all related character units and word units become activated within an interactive activation framework. Only one word ultimately wins the competition among the activated word units, and when this occurs, the word is identified and segmented from the upcoming character string simultaneously. Speed of word identification in the CRM is directly influenced by lexical characteristics such as word frequency and word length. Thus, the CRM can readily explain our word frequency findings for fixation times as high-frequency words received shorter fixation durations than low-frequency words. The visual and character units corresponding to a high-frequency word are activated significantly quicker and to a greater degree than the visual and character units corresponding to a low-frequency word. Furthermore, two-character words activate more related character and word units than one-character words, and therefore, it takes longer for two-character words to win the competition than one-character words, even when the visual complexity of one- and two-character words is quite similar. To be clear, based on our understanding, the CRM is readily able to explain the effects of length and frequency on lexical identification regardless of visual complexity. Furthermore, our findings demonstrate that each character is an important unit of information in Chinese reading. Words with more characters carry more information (i.e., are comprised of more units of information), and additional characters require additional time for processing with the amount of additional time, at some level, being unrelated to the visual complexity of those characters. To reiterate, while there have been a number of studies that have demonstrated the importance of (single and multiple character) word units in Chinese reading (e.g., Bai et al., 2008), the current study demonstrates that character representations are also important for word recognition during Chinese reading.

It is important to note, though, that not all our findings fit perfectly with the CRM. Perhaps a more difficult aspect of our results for the CRM to explain is the lack of interaction between frequency and word length for reading time measures on the target words. The CRM specifies that lexical characteristics such as length and frequency will directly and jointly influence the ease with which a word is identified. Within the interactive activation framework that sits at the heart of the CRM, both these variables should constrain lexical identification together and quite immediately. However, our results show that they do so independently. To our understanding, the additive pattern of these two factors is inconsistent with the CRM specifications.

What might explain the independent effects of word length and frequency? According to Sternberg’s (1969) additive factors logic, the additive effects of two variables in an experiment suggest that the variables influence separate processing stages. In contrast, interactive effects indicate that variables influence at least one common processing stage. Previous studies have shown that visual (e.g., stimulus quality) and lexical (e.g., frequency) factors additively affect word identification in isolated word-processing tasks (Yap & Balota, 2007; Yap et al., 2008).

Recently, Staub (2020) extended this work to examine similar effects in eye movements during natural reading. He conducted two substantial eye-tracking experiments in which the frequency and predictability of target words in sentences were factorially manipulated, while also manipulating visual contrast (Experiment 1) and font difficulty (Experiment 2). Staub’s study is related to the current experiment in that we also manipulated frequency across levels of visual complexity. Of course, here though, we were also able to manipulate word length without corresponding changes in visual complexity (i.e., increased word length in Staub’s study required extra letters that, unavoidably, concomitantly increased visual complexity). Staub’s results showed no reliable interactions between visual contrast and frequency or predictability, with reliable interactions between font difficulty and frequency or predictability only occurring in later eye movement measures. He concluded that his additive effects align with a two-stage account (Yap & Balota, 2007) in which manipulations of stimulus quality affect an initial perceptual stage of processing that occurs prior to lexical identification (the stage at which lexical characteristics such as frequency exert an influence).

Although there is clear value in the interpretative approach adopted by Staub (2020) in relation to his manipulations and findings, similar consideration of Additive Factors Logic in relation to the present results might not be entirely appropriate due to assumptions of thresholding and processing completion that are implicit in the logic. To be clear, adopting this approach in the interpretation of the current findings would require specification that word length and frequency constraints on the lexical identification process would necessarily affect different stages of processing (i.e., epochs of time). That is according to additive factors logic, for example, a stage of processing in which word length might constrain identification necessarily must be completed prior to initiation of processing in a stage during which frequency might constrain identification. It is difficult to understand how such temporally separate and independent influences might exist within the lexical identification mechanism associated with, for example, the CRM. Furthermore, it is very clear that during natural reading, many perceptual, linguistic, and oculomotor control processes take place simultaneously in time, that is, in a parallel, or, potentially, even in a cascadic manner. The application of Additive Factors Logic in these more complex (nuanced) processing systems might not be so straightforward (c.f., Schweickert et al., 2010), and for this reason, we do not appeal to it as directly as did Staub in relation to his work on effects of stimulus quality and frequency.^3^

All of this said, from our perspective, the current findings do have implications for how processing might take place through different stages involving different levels of representation. Recall, we found that Chinese readers spent more time initially processing two-character words than one-character words even when the visual complexity of those words was experimentally matched. These differences, therefore, are unlikely to derive from a perceptual stage of processing wherein a word’s visual complexity constrains lexical processing as per Staub’s findings. However, additional processing time associated with each additional character (regardless of the visual complexity of those characters), to us, suggests that the length manipulation does affect a stage of processing that occurs prior to processing associated with full lexical identification wherein influences of frequency are observed. The current findings for Chinese reading suggest a stage of processing associated with the linguistic units that comprise a word, that is, a level of processing associated with the characters of a word. Furthermore, processing associated with each linguistic unit, that is, each constituent character of a word, consumes additional time. The more characters that form a word, the more time is consumed, and importantly, our results show that at this level of processing, the visual complexity of a character has little influence on the amount of additional time that is required.

Thus, the metric that captures processing difficulty at this level is the number of linguistic units (i.e., the number of characters) comprising a word. If this suggestion is correct, then our results are of theoretical significance, providing evidence for processing of characters as abstract linguistic representations in and of themselves. That is, our results are suggestive of a stage of processing during Chinese reading that is associated with linguistic mental representations beyond visual characterisations, but representations that are not fully specified lexical representations at the level of the whole word. Processing of characters clearly consumes time, with words that have more characters requiring longer to process than words with fewer characters, and this difference cannot be attributed to processing required to encode a greater or reduced amount of visual information intrinsic to the stimulus. Note also that, implicit with this suggestion, changes in a word’s length in the absence of corresponding changes in visual complexity represent changes in a word’s linguistic complexity. That is to say, the processing cost here sits at a linguistic, not at a perceptual, level of representation. To be very clear, we consider that the present results provide strong evidence for linguistic processing of abstract character representations in word identification during natural Chinese reading. We see this as the most important theoretical implication of this experimental work.

In summary, our study provides the first well-controlled demonstration of word length and word frequency effects on eye movement control during natural Chinese reading. When visual complexity was controlled, the length of a word was shown to reliably affect both lexical identification and saccadic targeting, while word frequency was shown to solely influence lexical identification. More importantly, word length and word frequency independently influenced eye movement control during reading one- and two-character target words and these effects were not modulated by visual complexity. Some, but perhaps not all, aspects of these results fit neatly with the CRM, but most importantly, the current findings provide support for abstract character representations in word identification during natural Chinese reading.

## Notes

1. Of course, the visual complexity of a word depends, to some extent, on the particular letters that comprise it. For example, the letters “f,” “g” and “w,” are presumably more visually complex than the letters “l,” “c,” and “o,” in the sense that they have more constituent elements (perhaps like the strokes that comprise a character in Chinese). Thus, in principle, it may be possible that some longer words may be visually simpler than other, particularly, shorter words (e.g., perhaps “coin” might be less complex than “egg”). However, in the vast majority of cases, in alphabetic languages, longer words will be more visually complex than shorter words due to their increased number of constituent letters, each of which contributes to the overall visual complexity of the word.

2. Given that word length in the current study contains a number of comparisons across the three experimental conditions, we ran the models once more this time using the glht function of package multcomp in R to adjust p values and control for the multiple comparisons (Hothorn et al., 2008). There were no changes in the pattern of the significance. Furthermore, the type II Wald χ2 test was conducted using the Anova function of the car package (Version 3.1-2) in *R* (Fox et al., 2013) to examine the overall effects of word length, frequency, and their interactions. The results showed that there were reliable effects of word length on GD, TRT, MLP, and SP (all χ^2^ > 30.35, *p* < .01) and of frequency on all reading time measures (all χ^2^ > 11.74, *p* < .01) and MLP (χ^2^ = 5.17, *p* = .02). Critically, the interaction between the two conditions was not reliable in any of the measures (all χ^2^ < 3.21, *p* > .19). Overall, these results were entirely in accord with the results of our LMMs.

3. We are grateful to Sascha Schroeder for his incisive comments in relation to this point.
